# Contribution of Scanopelvimetry in the Prognosis of Childbirth

**DOI:** 10.7759/cureus.2936

**Published:** 2018-07-06

**Authors:** Lova Hasina Rajaonarison Ny Ony Narindra, Christian Tomboravo, Honjaniaina Rasolohery, Emmylou Prisca Gabrielle Andrianah, Gabriel Pierana Randaoharison, Ahmad Ahmad

**Affiliations:** 1 Medical Imaging, CHU Jra, Antananarivo, MDG; 2 Complexe Mère Enfant, CHU Pzaga, Antananarivo, MDG

**Keywords:** scanopelvimetry, magnin index, narrowed basin, childbirth mode

## Abstract

Objectives

To determine the frequency of the demand of radiopelvimetry in pregnant women and to assess the obstetrical prognosis.

Methods

Retrospective study about 19 months, including a total of 45 pregnant women who underwent scanopelvimetry to evaluate the biometry of the pelvis. Scanopelvimetry was performed from 36th week of pregnancy.

Results

The average age of the patients was 26.9 years. The frequency of the demand of scanopelvimetry was 0.97%. The height of the patients was greater than 150 cm in 84.44%. The indications of radiopelvimetry were dominated by the clinical suspicion of pelvic narrowing (57.78%), followed by the cephalopelvic confrontation (33.33%) and the cicatricial uterus (8.89%). Narrowed pelvis was observed in 28.89% of cases (13/45), with a Magnin index of less than 21. A caesarean section during labor was performed in 24.44% of the cases. No death of newborn was reported.

Conclusion

The frequency of the demand of scanopelvimetry is low but the rate of surgical pelvis is high among suspect cases.

## Introduction

Scanopelvimetry is an imaging technique used in obstetrics to explore the pelvic bone. It gives the possibility to examine the morphology of the pelvis and to realize quickly a reliable pelvimetry, and is economical in irradiation [1]. This work on the pelviscanner aims to improve maternal and child health. We propose in this study to determine the frequency of the demand of scanopelvimetry, to determine the frequency of the surgical pelvis and to assess the obstetrical prognosis.

## Materials and methods

**Figure 1:**
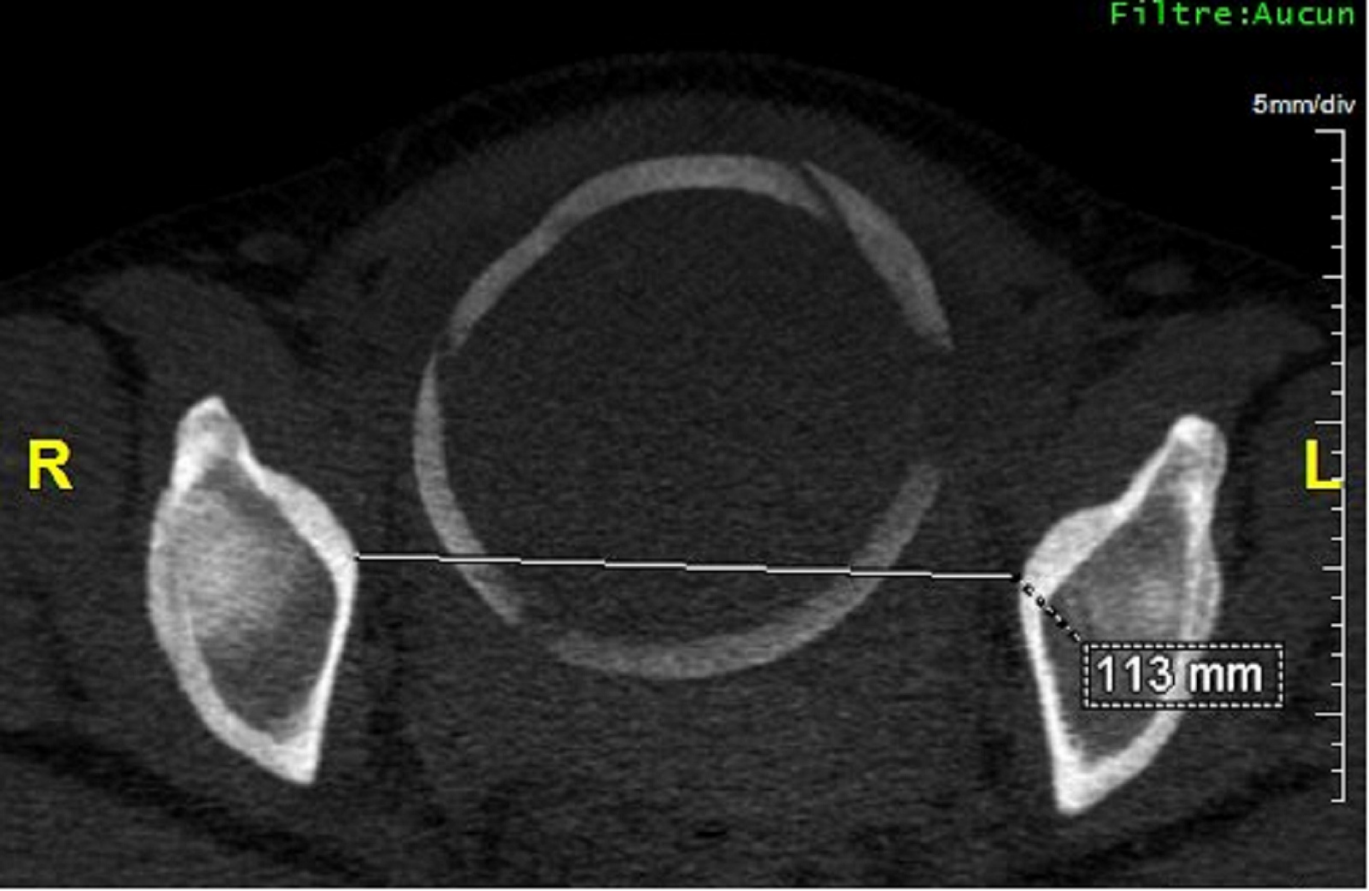
Scanopelvimetry, axial maximum intensity projection (MIP) reconstruction showing median transverse diameter.

**Figure 2:**
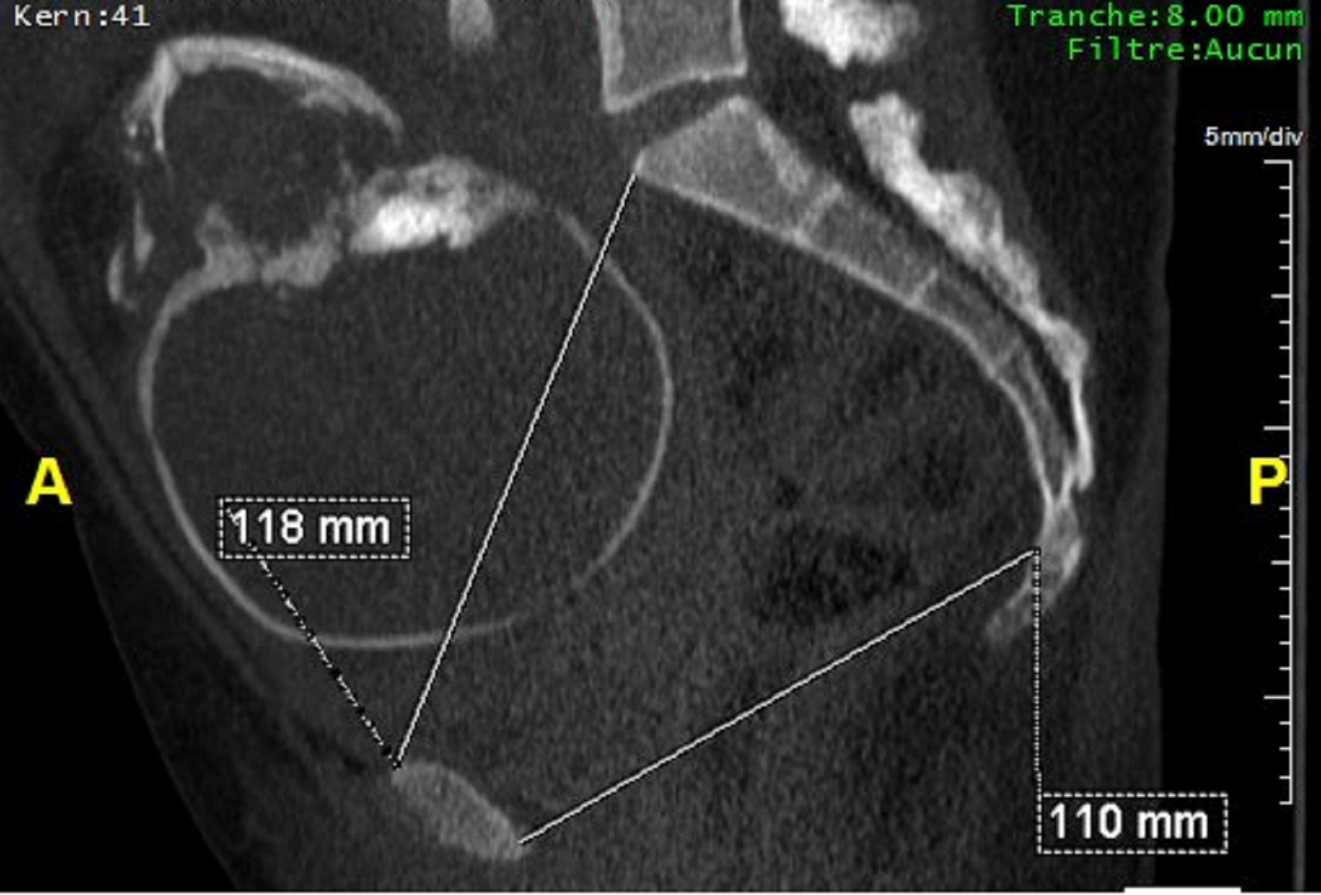
Scanopelvimetry, sagittal maximum intensity projection (MIP) reconstruction showing promonto-retro-pubic diameter and the sacro-sub-pubic diameter.

It was a descriptive retrospective study carried out at the Department of Medical Imaging of a polyclinic in Antananarivo (Madagascar) over a period of 19 months, from 1 August 2014 to 31 March 2016, and for which we had collected the files of patients on whom a computed tomography (CT) scan pelvimetry was performed, and who had a postpartum follow-up record. A predetermined survey sheet allowed to register the birth process and operative reports from the referring institutions. We measured the median transverse diameter (MT), the bi-sciatic diameter, the bi-ischiatic diameter, the promonto-retro-pubic diameter (PRP), the sacro-sub-pubic diameter, the rope and the rise of the sacrum (Figures 1, 2).

Thus, we calculated the Magnin index which is equal to the sum of the PRP and the MT diameters. The normal value of the Magnin index is greater than 23.

## Results

**Figure 3:**
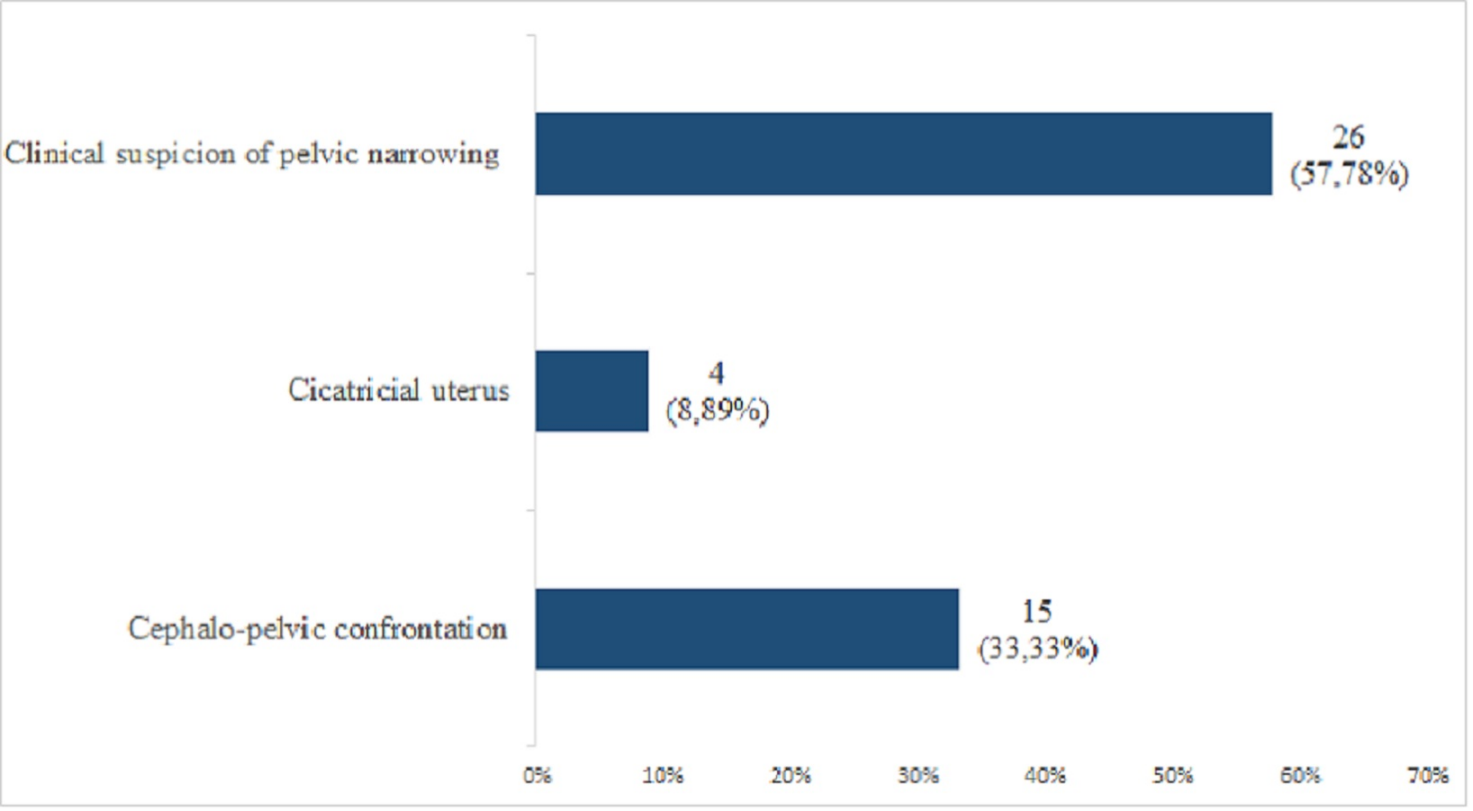
Distribution of patients according to the indication of pelviscanner.

We had collected 54 (0.97%) cases of scanopelvimetry on the 5552 scanners performed in the center during the study period, and had retained 45 cases because of no recording post-operative process. A total of 94.44% of the patients had a height greater than 150 cm. The average age of the patients was 26.9 years with an extreme ranging between 18 and 40 years. The indication of scanopelvimetry was dominated by the clinical suspicion of pelvic narrowing (57.77%) (Figure 3).

The Magnin index was less than 21 and between 21 and 23 in respectively 28.89% and 26.67% (Table 1).

**Table 1:** Distribution of patients according to the index of Magnin.

Magnin index	Number (n)	Percentage (%)	Conclusion of pelviscanner
20-21	13	28.89	Narrowed
21-23	12	26.67	Limited
23-26	20	44.44	Favorable
Total	45	100	-

The pelvis was narrowed in 28.89% of the cases.

The obstetric management was emergency caesarean section in 24.44% of cases and caesarean prophylaxis in 28.89% of the cases (Table 2).

**Table 2:** Distribution of patientes according to the mode of childbirth.

Mode of childbirth	Number (n)	Percentage (%)
Vaginal delivery	21	46.67
Caesarean during labor	11	24.44
Prophylactic caesarean	13	28.89
Total	45	100

A caesarean section was performed in 24 (53.33%) of the parturients, and 13 of them had a Magnin index of less than 21 (Table 3).

**Table 3:** Association between the Magnin index and the mode of childbirth.

		Mode of childbirth						Total	
		Vaginal delivery		Prophylactic caesarean		Caesarean during labor			
Magnin index		n	%	n	%	n	%	n	%
	20-21	-	-	13	28.89	-	-	13	28.89
	21-23	3	6.67	-	-	9	20	12	26.67
	23-26	18	40	-	-	2	4.44	20	44.44
Total		21	46.67	13	28.89	11	24.44	45	100

Two of our patients were caesarized while they had a Magnin index between 23 and 26. One for a refusal to push and the other for a caesarean of "convenience", i.e., at the request of the patient without medical or obstetrical indication.

No newborn deaths were reported in this study.

## Discussion

The frequency of scanopelvimetry was 0.97% of the scanners performed in our study whereas a French study showed a higher value reaching 5% [2]. This frequency is relatively low and could be explained by the high cost of the scanner, which is not within our grasp and because of the absence of a social security system compared to developed countries. An Australian study [3] showed a higher frequency (2.8%) than the present study, but weaker than the French study, while another African study showed a value of 1.02% [4], comparable to the present study.

Patients aged between 25 and 30 years were most represented, 35.56% of the cases. The mean age was 26.9 years, comparable to that of Cheikhani et al. [5] in Morocco, which had a mean age of 26.5 years, while in Munich, the mean age was higher estimated at 31.73 years according to Lenhard et al. [6]. This states that in developed countries, the age of onset of pregnancy is later.

Most of our patients (84.44%) were more than 150 cm in size, comparable to that of Adjenou et al. [4]. However, the study of Awonuga et al. in Georgia showed that the size of the parturient is not a sufficiently sensitive or specific factor to predict the permeability of the maternal pelvic bone [7], while more than half (55%) of maternity wards, in France, prescribe a pelvimetry in case of small size of the parturient according to Sataf et al. [8]. Scanopelvimetry was indicated in 57.78% of cases for a clinical suspicion of pelvic shrinkage, 33.33% for cephalo-pelvic confrontation and 8.89% for cicatricial uterus. According to Adjenou et al. [4], the clinical suspicion of pelvic narrowing accounts for 48% of the indications for scanopelvimetry. As regards the practice of scanopelvimetry in the case of a history of cicatricial uterus, our result is close to that of Peultier et al. [9] who found 9.4% of the indications.

The principle of scanopelvimetry in women who had a previous caesarean section during the labor at the previous pregnancy would not unnecessarily repeat a test of labor to women who have a narrowed pelvis, and thus indirectly to avoid predictable uterine ruptures. However, according to the 2012 recommendations of the French College of Gynecologists and Obstetricians (CNGOF), radiopelvimetry is not necessary to decide the delivery route and to conduct labor in the event of vaginal delivery attempt after a previous caesarean section [10].

In our study, the Magnin index was less than 21 in 28.89%, between 21 and 23 in 26.67% and greater than 23 in 44.44% whereas a French study found 55.5% of unfavorable prognosis according to the Magnin index (<23 cm) [11]. However, our result is close to that of Christian et al., who found 33.33% of narrowed pelvis [12] but widely different than that obtained by Adjenou et al. who found 83% of narrowed pelvis. In this study, the obstetrical management was emergency caesarean in 24.44% of cases and prophylactic caesarean in 28.89% of cases, which is comparable to that found by Yamani and Rouzi in Saudi Arabia with respectively 23% and 28% [13] of cases. All patients with narrowed pelvis (28.89%) were caesarized as in the study of Adjenou et al. [4]. Among the caesarized patients, 54.17% had a Magnin index of less than 21 and 37.5% had between 21 and 23. No patient gives birth by vaginal delivery with a Magnin index below 21, as in the study of Frémondière and Fournie [11], which would authenticate the importance of this index in the prognosis of childbirth.

In our study, 12 parturients had a limit basin and nine (75%) of them were caesarized, which approximates the result of Cissé et al. [14] who reported 148 cases of surgical basins leading to a prophylactic caesarean section, 296 cases of limit pelvis, of which 148 (73.6%) cases were subjected to a labor test leading to childbirth by vaginal delivery. This explains the importance of scanopelvimetry, which allows the prophylactic caesarean to spare the newborn from complications such as acute fetal distress or even in utero fetal death.

However, some authors find that knowledge of the pelvis measurements has a negative role and influences the practitioner towards the prophylactic caesarean section or during labor [15], others proclaim that knowledge of the basin as narrowed is among the factors most linked to the failure of the labor test.

## Conclusions

The frequency of the demand of scanopelvimetry is low but the rate of surgical pelvis detected is high among the suspected cases. Childbirth involves maternal and/or fetal risk if pelvic permeability is not safe. The practice of scanopelvimetry helps to minimize this risk.
